# Association of mental health, quality of life, and SARS-CoV-2 infection in individuals in need of care: Results from a multicentre registry study

**DOI:** 10.1371/journal.pone.0323017

**Published:** 2025-05-09

**Authors:** Karoline Lukaschek, Heidi Hentschel, Marietta Rottenkolber, Martin Alberer, Susanne Winter, Maria Sebastia͂o, Florian Arend, Tobias Dreischulte, Ildikó Gágyor, Anita Hausen, Michael Hoelscher, Christian Janke, Thomas Kühlein, Daniel Teupser, Jochen Gensichen, for the BACOM Study Group

**Affiliations:** 1 Institute of General Practice and Family Medicine, University Hospital, LMU Munich, Munich, Germany; 2 Division of Infectious Diseases and Tropical Medicine, Medical Centre of the University of Munich (LMU), Germany; 3 Institute of General Practice, Friedrich-Alexander-University of Erlangen-Nuremberg, Erlangen, Germany; 4 Institute of Laboratory Medicine, University Hospital, LMU Munich, Munich, Germany; 5 Department of General Practice, University Hospital Würzburg, Würzburg, Germany; 6 Katholische Stiftungshochschule München, University of Applied Sciences, Campus Munich, Faculty of Health and Nursing, Munich, Germany; Johns Hopkins Bloomberg School of Public Health: Johns Hopkins University Bloomberg School of Public Health, UNITED STATES OF AMERICA

## Abstract

**Objective:**

Investigating the association between mental health, quality of life, and SARS-CoV-2 infection in individuals in need of care compared to independent living individuals. Individuals in need of care include both care home residents and those receiving care either through an outpatient care service or from family members.

**Methods:**

This cross-sectional study assessed symptoms of depression (PHQ-9 > 9) and anxiety (GAD-7 > 9), quality of life (EQ-5D-5L, EQ-VAS), dementia (SIS), SARS-CoV-2 infection and socio-demographic

variables in the total sample (N = 978, 64.4% female, mean age: 77.5 ± 13.8 years) and subgroups (study group, STG, n = 532, individuals in need of care, SARS-CoV-2 positive; control group 1, CG1, n = 213, individuals in need of care, SARS-CoV-2 negative; control group 2, CG2, n = 233, independent living individuals, SARS-CoV-2 positive). Multivariate logistic regressions were performed.

**Results:**

Depressive symptoms (PHQ-9 > 9) were significantly associated with lower quality of life in the total sample (EQ-VAS: OR 0.96, 95% CI 0.95–0.97, p < 0.001; EQ-5D-5L: OR 0.14, 95% CI 0.07–0.29, p < 0.001) and across all subgroups. Anxiety (GAD-7 > 9) was significantly associated with lower quality of life in the total sample (EQ-VAS: OR 0.97, 95% CI 0.95–0.98, p < 0.001; EQ-5D-5L: OR 0.19, 95% CI 0.08–0.50, p < 0.001) and all subgroups except CG1. In individuals in need of care with COVID-19, depressive symptoms were additionally associated with symptomatic infection (OR 3.47, 95% CI 1.45–8.28, p = 0.005).

**Conclusion:**

Depression and anxiety were significantly associated with reduced quality of life, irrespective of living environment or SARS-CoV-2 infection status, underscoring the need for targeted mental health interventions in older adults. While our model explained a considerable portion of the variability in depression and anxiety, further research is needed to account for the remaining proportion.

## Introduction

The COVID-19 pandemic profoundly affected physical and mental well-being worldwide [1,2].

Beyond the direct health risks, the fear of an unfamiliar and severe illness, coupled with restrictive containment measures such as quarantine and lockdowns, created significant psychological distress [3]. Among the most affected were older adults, who not only faced higher morbidity and mortality risks but also endured prolonged social isolation due to contact restrictions and limited access to digital communication alternatives [4].

While research has shown a notable increase in depression among older adults during the pandemic [5], evidence also suggests that the overall mental well-being of the older general German population remained largely stable during the COVID-19 lockdowns [6]. However, this applies only to older adults living at home and does not extend to those in need of care or residing in nursing homes [7,8]. In Germany, a person is defined as being in need of (long-term) care if they are unable to independently manage or cope with physical, mental, cognitive or health-related demands or burdens in the long term - all of which are of greater or considerable severity [9]. At the beginning of 2018, around 3.3 million people were affected, almost two-thirds of them women and the majority (81.5%) was aged 60 or older [10]. Demographic trends are expected to lead to a further increase in the coming decades, with a forecast of approximately 7 million by 2050 [11].

During the pandemic, individuals in need of care were quickly recognized as particularly vulnerable and subject to prolonged and intense contact restrictions, social distancing, quarantine measures, and testing requirements [12]. Despite older populations showing resilience [6,13], daily life still posed significant challenges for nursing home seniors, especially during high-incidence periods. Necessary medical visits were often postponed or cancelled due to infection fears and healthcare focus on COVID-19 patients [14]. Beneficial social activities were halted, leading to stress reactions (in terms of changes in blood values and disruption of homeostasis) and mental ill-health such as depression in the isolated individuals [15,16].

Despite extensive research on the impact of COVID-19 measures and SARS-CoV-2 infection on the mental health of older adults, particularly anxiety disorders and depression, there is limited knowledge about how mental health and quality of life correlate with the severity of SARS-CoV-2 symptoms. This study is the first to the association between mental health, health-related quality of life, and the severity of a SARS-CoV-2 infection in individuals in need of care tested positive for SARS-CoV-2 compared to a) individuals in need of care not tested positive for SARS-CoV-2 and b) independent living individuals tested positive for SARS-CoV-2. By comparing these groups, the study provides critical insights for developing targeted mental health interventions in older adults.

## Materials and methods

### Study design

The Bavarian Outpatient Covid-19 Monitor (BaCoM) was a multi-centre, open registry study, conducted from 1 January 2021–31 December 2023 under the direction of the Institute of General Medicine, Hospital of the LMU Munich. Funding was provided by the Bavarian State Ministry of Health, Care and Prevention until 31 December 2023. A detailed description of the study design has already been published elsewhere [17].

The study was conducted in accordance with the guidelines of the Declaration of Helsinki [18]. Participants were recruited from 1 March 2021–31 August 2023. Signed informed consent from the participant or a legal guardian was obtained by research assistants. The ethics committee of the LMU Munich approved of the study on 26 February 2021. The study was registered in the German Clinical Trials Register (DRKS00026039).

### Recruitment

Participants were recruited throughout Bavaria via general practitioners (GPs) and care facilities. A research assistant informed the participants on site, obtained written consent, and ideally carried out the first interview, including taking blood samples and measuring vital signs. Staff from nursing homes, caregiving relatives and GPs were also interviewed, with the latter being actively involved in the study if they were interested. Thus, in addition to their participation in the patient survey, caregiving relatives were given separate questionnaires addressing their role and needs. GPs also played a key role, not only in recruiting potential participants but also in providing insights into their own experiences and concerns

### Inclusion and exclusion criteria for participation in the study

Inclusion criteria for the STG were a proven SARS-CoV-2 infection and an existing need for care/support, which was defined either by the existence of a care level (1–5) or according to the assessment of the GP or the study assistant using the frailty scale [19]. On this nine-level categorisation, a person is classified as “mildly fragile” from level 5 and therefore at least in need of support. A more in-depth description of the inclusion criteria has been published previously [20]. People with terminal illnesses were excluded due to their low remaining life expectancy (<six months). Further exclusion criteria for all groups were young age (< 18 years) or refugee status.

### Study population

The study group (STG, n = 532) consists of individuals in need of care who had tested positive for SARS-CoV-2 since 1 March 2020. Individuals in control group 1 (CG1, n = 213) are those in need of care who had not yet tested positive for SARS-CoV-2 by the time of the survey. Control group 2 (CG2, n = 233) consists of independent living individuals with a positive SARS-CoV-2 test.

### Measures

Depression was assessed using the Patient Health Questionnaire (PHQ)-9, a 9-item self-report questionnaire which assesses depressive symptoms in the last 2 weeks [21]. Items are rated on a 4-point Likert-type scale, ranging from 0 (not at all) to 3 (nearly every day). The total score can range from 0 to 27, with high scores meaning high depression. Based on the original validation studies, the total score can then be interpreted as suggesting no depression (0–4), mild (5–9), moderate (10–14), moderately severe (15–19), or severe (20–27). A cut off score of 10 is suggested as indicating a possible diagnosis of a major depressive disorder [22]. Generalized Anxiety Disorder (GAD) was assessed using the Generalized Anxiety Disorder 7-item scale (GAD-7), a self-report questionnaire consisting of 7 items, which inquiries about the frequency of anxiety symptoms experienced over the past 2 weeks [23]. Participants rate each item on a 4-point Likert-type scale, ranging from 0 (not at all) to 3 (nearly every day). The total score ranges from 0 to 21, with higher scores indicating higher levels of anxiety. Consistent with the original validation studies, the total score can then be interpreted as suggesting minimal anxiety (0–4), mild (5–9), moderate (10–14), and severe (15–21) levels of anxiety. A cut-off score of 10 is commonly used to indicate a possible diagnosis of generalized anxiety disorder [24].

The Six-Item-Screener (SIS) is a brief cognitive screening tool used to quickly assess cognitive impairment in older adults [25]. It consists of six questions covering areas such as memory and orientation. A value <4 let to interviews with professional carers (e.g., nurses, GPs) or caregiving relatives.

We assessed health-related quality of life using the European Quality of Life 5 Dimensions 3 Level Version (EQ-5D-5L) questionnaire [26]. Participants rated their level of impairment in each of its five dimensions (mobility, self-care, usual activities, pain/discomfort, and anxiety/depression) on a five-level scale. Using an algorithm provided on the website (https://euroqol.org/), a score between 0 (very poor quality of life) and 1 (best possible quality of life) was calculated from the resulting five individual values. Additionally, we used the European Quality of Life visual analogue scale (EQ-VAS), where participants provide a self-rated assessment of their current health status on the day of the survey by placing a mark on a line ranging from 0 to 100 [27]. On this scale, 0 represents the worst imaginable state of health, while 100 represents the best imaginable state of health. Therefore, higher values indicate better perceived health, whereas lower values indicate poorer perceived health.

In addition, socio-demographic data, health status, symptoms in connection with SARS-CoV-2 and, if applicable, care-specific parameters (e.g., care level, setting) were recorded.

### Severity of COVID-19 infection

The severity COVID-19 was determined based on the hospitalisation of the patient: outpatient treatment corresponds to a mild course of the infection and hospitalisation to a moderate course [28]. If the patient had to be treated in an intensive care unit, a severe course was assumed. In addition, we recorded whether SARS-CoV-2 was symptomatic or asymptomatic.

### Statistical analysis

All analyses were performed with IBM SPSS Statistics, version 29.0.1.0. A p-value of less than 0.05 was considered significant.

Continuous variables were tested for normal distribution using the Shapiro-Wilk and Kolmogorov-Smirnov tests. With continuous values < 0.001, a normal distribution could not be assumed for any of the variables. The Spearman correlation test was used to analyse the potential correlations between the independent variables examined.

The dependent variables depression and anxiety were dichotomised (“depression yes/no”; “anxiety disorder yes/no”) using a cut-off at 10 points for each. Descriptive analyses were performed for the entire study population and according to group membership. If the distribution was not normal, the tables show the median, Q1 and Q3. In the case of a normal distribution, the mean and standard deviation were specified. Categorical variables are presented in absolute numbers and percentages. Logistic regression analyses were performed for our whole population and separately for each group using a backward stepwise entry method and LR (Likelihood Ratio) criterion.

All potential predictors, i.e., sex, age, BMI, education status, cognition according to the Six-Item-Screener, marital status, smoking status, EQ-5D-5L, EQ-VAS, symptomatic SARS-CoV-2 infection, severity by hospitalization were initially included in the model and then variables were gradually removed in case of p-value > 0.05. The variables “smokers” and “cognition according to the Six-Item-Screener” were not included in individual group analyses due to insufficient numbers.

Study participants with missing variables were excluded from the analysis, as there was often no information available on several of the parameters we analysed. Multiple imputations were not performed, resulting in n = 792 participants with valid data on anxiety and n = 804 participants with valid data on depression.

## Results

### Descriptive analyses

Results from the descriptive analysis of socio-economic data are shown in Table 1.

**Table 1:** Socio-economic Data of Study Participants.

	Total	Study Group	Control Group 1	Control Group 2
	n = 978	n = 532 (54,4%)	n = 213 (21,8%)	n = 233 (23,8%)
Sex; n (%)				
Female	630 (64,4)	364 (68,4)	147 (69,0)	119 (51,1)
Male	346 (35,4)	167 (31,4)	65 (30,5)	114 (48,9)
Age in Years				
Mean (SD)	77,5 (13,8)	80,4 (12,4)	83,0 (8,6)	66,1 (14,0)
Median	81,0	84,0	84,0	69,0
(Q1; Q3)	(71,0; 87,0)	(75,0; 88,0)	(78,0; 88,0)	(58,0; 76,0)
Age grouped in Years; n (%)				
< 60	105 (10,7)	33 (6,2)	3 (1,4)	69 (29,6)
60–69	111 (11,4)	49 (9,2)	6 (2,8)	56 (24,1)
70–79	227 (23,2)	105 (19,7)	56 (26,3)	66 (28,3)
80–89	378 (38,7)	231 (43,4)	105 (49,3)	42 (18,0)
> 90	145 (14,8)	104 (19,6)	41 (19,3)	- (-)
Marital Status; n (%)				
Living alone	584 (59,7)	369 (69,4)	147 (69,0)	68 (29,2)
Living together	380 (38,9)	154 (29,0)	61 (28,6)	165 (70,8)
Highest Education; n (%)				
None	21 (2,2)	16 (3,0)	5 (2,4)	- (-)
Primary/Elementary School	442 (45,2)	258 (48,5)	108 (50,7)	76 (32,6)
Secondary School	258 (26,4)	138 (25,9)	61 (28,6)	59 (25,3)
High School Diploma	233 (23,8)	101 (19,0)	34 (16,0)	98 (42,1)
Care Level; n (%)				
1	59 (7,9)*	42 (7,9)	17 (8,0)	–
2	173 (23,2)*	111 (20,9)	62 (29,1)	–
3	175 (23,5)*	134 (25,2)	41 (19,3)	–
4	65 (8,7)*	50 (9,4)	15 (7,0)	–
5	22 (3,0)*	16 (3,0)	6 (2,8)	–
Type of Care; n (%)				
Ambulant Care	282 (37,9)*	203 (38,2)	79 (37,1)	–
Inpatient Care	359 (48,2)*	252 (47,4)	107 (50,2)	–

Missing Values n (%): Sex = 2 (0,2); Age = 12 (1,2); Marital Status = 14 (1,4); Highest Education = 24 (2,5%); Care Level = 251 (33,7)*; Type of Care = 104 (14,0)*.

*n=745, Control Group 2 not applicable.

A total of 978 patients were included (mean age: 77.5 years, SD = 13.8, range: 19–103 years) in our analysis. The majority was female (n = 630, 64.4%). Patients in CG1 (n = 213) were slightly older (mean age: 83.0, SD = 8.6, range: 42–103 years), patients in CG2 (n = 233) slightly younger (mean age: 66.1 years, SD = 14.0, range: 19–89 years) than those in the STG (n = 532, mean age: 80.4 years, SD = 12.4, range: 24–103 years). Notably, in contrast to the other two groups, the majority of respondents in CG2 had a high school diploma or vocational diploma (n = 98; 42.1%), and there were no participants without a high school diploma.

Results of the descriptive analyses regarding health-related date are shown in Table 2. In general, the population we studied was slightly overweight (BMI 25.9 kg/m^2^) and predominantly non-smokers or ex-smokers (n = 874; 89.4%). The median value of the PHQ-9 was 4.00 points (Q1 = 2.00 points, Q3 = 7.00 points), with 15.3% (n = 150) of participants scoring ten or more points (range: 0–25 points), which corresponds to moderate or severe depression. The median value of the GAD-7 was 1.00 points (Q1 = 0.00 points, Q3 = 4.00 points), only 7.0% (n = 68) of participants achieved ten or more points (range: 0–21 points). Cognitive impairment as measured by the SIS was observed in 9.8% (n = 96) of all participants.

**Table 2:** Health-related Data of Study Participants.

	Total	Study Group	Control Group 1	Control Group 2
	n = 978	n = 532 (54,4%)	n = 213 (21,8%)	n = 233 (23,8%)
BMI in kg/m2				
Median	25,9	26,2	25,5	25,1
(Q1; Q3)	(22,9; 29,6)	(23,0; 30,2)	(22,5; 29,0)	(22,9; 29,0)
BMI in kg/m2, grouped; n (%)				
≥ 18,5	26 (2,7)	12 (2,3)	11 (5,2)	3 (1,3)
18,5–24,9	367 (37,5)	176 (33,1)	80 (37,6)	111 (47,6)
25,0–29,9	317 (32,4)	178 (33,5)	69 (32,4)	70 (30,0)
30,0–34,9	145 (14,8)	85 (16,0)	25 (11,7)	35 (15,0)
35,0–39,9	51 (5,2)	31 (5,8)	11 (5,2)	9 (3,9)
≤ 40,0	16 (1,6)	11 (2,1)	4 (1,9)	1 (0,4)
Smoker; n (%)				
Yes	66 (6,8)	35 (6,6)	14 (6,6)	17 (7,3)
No or Former Smoker	874 (89,4)	471 (88,5)	190 (89,2)	213 (91,4)
Cognition according to SIS; n (%)				
> 3	822 (84,1)	430 (80,8)	163 (76,5)	229 (98,3)
< 4	96 (9,8)	68 (12,8)	28 (13,2)	- (-)
EQ-5D-5L				
Median	0,81	0,73	0,71	0,97
(Q1; Q3)	(0,51; 0,94)	(0,41; 0,88)	(0,43; 0,89)	(0,91; 1,00)
EQ-VAS				
Median	65,0	60,0	60,0	80,0
(Q1; Q3)	(50,0; 80,0)	(50,0; 80,0)	(50,0; 80,0)	(70,0; 90,0)
Symptoms; n (%)				
Yes	626 (81,8)*	408 (76,7)	–	218 (93,6)
No	126 (16,5)*	111 (20,9)	–	15 (6,4)
Severity by Hospitalization; n (%)				
Outpatient	478 (62,5)*	409 (76,9)	–	67 (28,8)
Regular Ward	50 (6,5)*	50 (9,4)	–	- (-)
Intensive Care Unit	23 (3,0)*	20 (3,8)	–	3 (1,3)
GAD-7				
Median	1,00	2,00	1,00	1,00
(Q1; Q3)	(0,00: 4,00)	(0,00; 4,00)	(0,00; 4,00)	(0,00; 3,00)
GAD-7 > 9 Points; n (%)	68 (7,0)	45 (9,2)	16 (7,5)	7 (3,0)
PHQ-9				
Median	4,00	4,00	4,00	3,00
(Q1; Q3)	(2,00; 7,00)	(2,00; 8,00)	(2,00; 9,00)	(1,00; 5,00)
PHQ-9 > 9 Points; n (%)	150 (15,3)	91 (17,1)	36 (16,9)	23 (9,9)

Missing Values: BMI = 56 (5,7); Smoking Status = 38 (3,9); Cognition according to SIS = 60 (6,1); EQ-5D-5L = 45 (4,6); EQ-VAS = 55 (6,7); Symptoms = 13 (1,3); Severity by Hospitalization = 214 (28,0); GAD-7 = 66 (6,8); PHQ-9 = 55 (5,6).

* n=765, Control Group 1 not applicable.

In the examination of health-related quality of life, a median value of 0.81 (Q1 = 0.51, Q3 = 0.94) was achieved for the EQ-5D-5L and 65.0 (Q1 = 50.0, Q3 = 80.0) for the EQ-VAS. Notably, we found higher EQ-5D-5L and EQ-VAS health-related quality of life scores in the independent CG2 (EQ-5D-5L: 0.97 (Q1 = 0.91, Q3 = 1.00) vs. 0.73 (Q1 = 0.41, Q3 = 0.88) in SG and 0.71 (Q1 = 0.43, Q3 = 0.89) in CG1; EQ-VAS: 80.0 (Q1 = 70.0, Q3 = 90.0) vs. 60.0 (Q1 = 50.0, Q3 = 80.0) in both STG and CG1).

### Logistic regression analyses

There was a strong correlation between EQ-5D-5L and EQ-VAS (0.527 according to Spearman) as well as between SARS-CoV-2 symptoms and severity by hospitalization (0.689 according to Spearman). In each case, the correlation was significant at the 0.01 level (2-sided).

As shown in Table 3, after conducting backward logistic regression analysis, several variables were found to be significantly associated with the likelihood of higher PHQ-9 scores, indicating a tendency toward depression. Specifically, age in years (OR: 0.98, 95% CI 0.96–0.99 p value 0.014), EQ-5D-5L (OR: 0.14, 95% CI 0.07–0.29, p value <0.001) and EQ-VAS score (OR: 0.96, 95% CI 0.95–0.97, p value <0.001) were identified as significant predictors. We achieved a Nagelkerke R² of 0.285. The same analysis was conducted for the GAD-7, with results shown in Table 3. Significant associations were found for the EQ-5D-5L (OR: 0.19, 95% CI 0.08–0.50, p value <0.001) and the EQ-VAS score (OR: 0.97, 95% CI 0.96–0.99, p value <0.001) with a Nagelkerke R² of 0.174.

**Table 3:** Logistic Regression ‘Backward LR’ PHQ-9 and GAD-7 total sample.

	PHQ-9	
	Total*	
	OR [95%KI]	p-Value
Age in Years	0,980 [0,964-0,996]	0,014
BMI in kg/m2	0,965 [0,930-1,002]	0,065
EQ-5D-5L	0,137 [0,065-0,290]	< 0,001
EQ-VAS	0,959 [0,948-0,970]	< 0,001
Symptoms	1,594 [0.976-2.604]	0,063
	GAD-7	
	Total**	
	OR [95%KI]	p-Value
EQ-5D-5L	0,194 [0,076-0,497]	< 0,001
EQ-VAS	0,971 [0,955-0,986]	< 0,001
Symptoms	1,970 [0,995-3,898]	0,052

*Missing Values: Total n=174; ** Missing Values: Total n=186.

Results of the logistic regression analysis by group are shown in Table 4 (PHQ-9) and Table 5 (GAD-7).

**Table 4:** Logistic Regression ‘Backward LR’ PHQ-9 by groups.

	Study Group		Control Group 1		Control Group 2	
	OR [95%KI]	p-Value	OR [95%KI]	p-Value	OR [95%KI]	p-Value
Age in Years	0,985 [0,964-1,006]	0,161	1,013 [0,962-1,067]	0,618	0,956 [0,924-0,989]	0,009
EQ-5D-5L	0,098 [0,040-0,242]	< 0,001	0,233 [0,059-0,909]	0,036	0,243 [0,010-5,969]	0,387
EQ-VAS	0,967 [0,953-0,981]	< 0,001	0,972 [0,951-0,992]	0,008	0,922 [0,895-0,950]	< 0,001
Symptoms	3,467 [1,451-8,281]	0,005	–	–	1,639 [0,129-20,765]	0,703

Missing Values: Study Group n  =  80; Control Group 1 n  =  40; Control Group 2 n  =  9.

**Table 5:** Logistic Regression ‘Backward LR’ GAD-7 by groups.

	Study Group		Control Group 1		Control Group 2	
	OR [95%KI]	p-Value	OR [95%KI]	p-Value	OR [95%KI]	p-Value
Age	0,971 [0,946-0,996]	0,023	1,026 [0,273-1,542]	0,544	1,008 [0,938-1,085]	0,823
EQ-5D-5L	0,198 [0,055-0,604]	0,004	0,671 [0,361-4,946]	0,706	0,303 [0,001-80,038]	0,675
EQ-VAS	0,966 [0,949-0,984]	<0,001	0,978 [0,951-1,005]	0,104	0,960 [0,924-0,999]	0,043
Marital Status	1,794 [0,873-3,688]	0,112	1,336 [0,361-4,946]	0,664	0,216 [0,038-1,248]	0,087

Missing Values: Study Group n  =  89; Control Group 1 n  =  38; Control Group 2 n  =  12.

Higher PHQ-9 scores in the STG were significantly associated with lower health-related quality of life in the EQ-5D-5L (OR: 0.09, 95% CI 0.04–0.24, p value <0.001) and EQ-VAS (OR: 0.97, 95% CI 0.95–0.98, p value <0.001) as well as a symptomatic COVID-19-infection (OR: 3.47, 95% CI 1.45–8.28, p value 0.005). For CG1, there was also a significant influence of EQ-5D-5L (OR: 0.23, 95% CI 0.06–0.91, p value 0.036) and EQ-VAS (OR: 0.97, 95% CI 0.95–0.99, p value 0.008). In CG2, age in years (OR: 0.96, 95% CI 0.92–0.99, p value 0.009) and the EQ-VAS (OR: 0.92, 95% CI 0.90–0.95, p value <0.001) were significant factors.

We achieved a Nagelkerke R² of 0.298 in the STG, 0.166 in CG1 and 0.353 in CG2.

A reduced health-related quality of life determined by EQ-5D-5L (OR: 0.20, 95% CI 0.06–0.60, p value 0.004), EQ-VAS (OR: 0.96, 95% CI 0.95–0.98, p value <0.001), and age (OR: 0.97, 95% CI 0.95–0.99, p value 0.023) are significantly associated with higher GAD-7 values in the STG. In CG2, the EQ-VAS appeared to be the only significantly influencing variable (OR: 0.96, 95% CI 0.92–0.99, p value 0.043).

We achieved a Nagelkerke R² of 0.215 in the STG, 0.039 in CG1 and 0.157 in CG2.

## Discussion

A key finding is that depressive symptoms were significantly associated with lower quality of life across all groups, irrespective of SARS-CoV-2 infection status. This suggests that broader structural factors—such as social isolation, loss of autonomy, and access to care—may be more influential in determining mental health outcomes than the infection itself. As recommended by the EuroQol group, we used both components of the EQ-5D questionnaire: the index and the EuroQol Visual Analogue Scale (EQ-VAS). While both measures are significantly correlated and reliable for evaluating health-related quality of life, the EQ-VAS appears particularly well-suited for assessing the perceived health status of older individuals [29].

Furthermore, anxiety was more strongly associated with quality of life among SARS-CoV-2-positive individuals, indicating that psychological distress related to COVID-19 may persist even after recovery. These findings underscore the need for targeted mental health interventions in long-term care settings, where pandemic-related restrictions may have had a disproportionate impact on emotional well-being. Future research should explore whether strategies such as increased psychosocial support, structured social interactions, and enhanced access to mental health care can mitigate these effects and improve quality of life for older adults in institutional settings.

Significantly more women than men participated in our study. Women are more willing to provide information and are therefore more willing to talk about (psychological) problems [30]. Moreover, women have a higher life expectancy and thus, are likely overrepresented - especially in the cohorts born between 1920 and 1930 - due to the countless deaths of men during the Second World War [31,32].

In our total sample, we found a significant association between BMI and depression. On average, participants in our study were slightly overweight with a median of around 26 kg/m^2^. Previous studies have shown a positive correlation between BMI and depressive symptoms [33,34] Several mechanisms have been proposed to explain the link between BMI and depression: Obesity is thought to contribute to depression via negative self-image or impaired physical function [35], while conversely, depression may lead to weight gain and obesity via unhealthy behaviour, poor sleep quality, and side effects of anti-depressant medication. Particularly among older adults, the association of BMI and depressive symptoms seems to be bidirectional [36].

The significant association of older age and higher levels of depression apparent in our data is well documented in the literature [37,38]. Depression in older age is typically associated with losses (e.g., health and mobility, social networks and significant others through bereavement). Due to the Covid-19 pandemic, new factors have emerged, e.g., stress-related factors and feelings or worries related to the pandemic, factors directly related to COVID-19 (e.g., having infected relatives/friends) and factors related to the measures that were taken to reduce the spread of COVID-19 (e.g., containment measures, difficulty receiving medical care and difficulty obtaining medications) [5].

The differences in health-related quality of life between the participants in need of care (SG, CG1) and participants who were still independent (CG2) were striking. The medians for both the EQ-5D-5L (0.72 versus 0.97) and the EQ-VAS (60.0 versus 80.0) diverged significantly. With regard to SARS-CoV-2 infection, we found that more subjects in SG2 than in the SG reported symptomatic illness. This could be related to the fact that more people in this group felt that their course was severe and thus, agreed to take part in the study, or were encouraged to do so by their GP.

We found a significance association between depression and low quality of life through all subgroups,

which means irrespective of need of care or SARS-CoV-2 positivity. This might be explained by the loss of autonomy and independence [39] but also by experiences related to the SARS-CoV-2 infection [5].

The association of anxiety disorder and low quality of life we found in participants who were tested positive for SARS-COV-2 irrespective of their care status (STG and CG2) might be triggered by Covid-19 in general [40] explained by the psychological stress which often occurs after intensive medical treatment, especially when patients are experiencing feelings of helplessness and vulnerability, e.g., in the intensive care unit [41,42]. In our study, 20 individuals from the SG were treated in the intensive care unit, but only 3 from CG2. Thus, it was not possible to generate sufficient data for comparisons.

Overall, this study provides novel insights into the persistent impact of mental health on quality of life in older adults, regardless of living situation or COVID-19 infection. The findings highlight the urgent need for mental health interventions tailored to older individuals, particularly those in long-term care.

### Strengths and limitations

The strength of this comprehensive study lies in the inclusion of a substantial number of older patients from various social classes and backgrounds, who are rarely interviewed in detail and in person. The study also included subjects with cognitive impairment, with necessary information from proxies when needed. However, proxy responses may not accurately reflect patients’ opinions or conditions.

The study has limitations that need to be addressed: Due to the cross-sectional nature of our study, it is not possible to determine the temporal relationship between depression and the need for permanent care or between COVID-19 infections and mental health. Regarding the assessment of medical care in the context of SARS-CoV-2 infection, the questionnaire did not always clarify if patients were treated as out- or inpatients, and reliable retrospective data was sometimes unattainable.

Due to the specific population studied, the generalisability of the results is limited. Finally, while our model explained a considerable portion of the variability in depression and anxiety, further research is needed to account for the remaining proportion.

## Conclusion

Depression and anxiety were significantly associated with reduced quality of life, irrespective of living environment or SARS-CoV-2 infection status, underscoring the need for targeted mental health interventions in older adults. With the rising number of people needing care, further studies should examine if low-threshold improvement in quality of life also improves mental health. Additionally, maintaining independence as long as possible and reducing the stigma around care dependency seem warranted.
